# Selective Estrogen Receptor Modulators: Cannabinoid Receptor Inverse Agonists with Differential CB1 and CB2 Selectivity

**DOI:** 10.3389/fphar.2016.00503

**Published:** 2016-12-22

**Authors:** Lirit N. Franks, Benjamin M. Ford, Paul L. Prather

**Affiliations:** Department of Pharmacology and Toxicology, University of Arkansas for Medical Sciences, Little RockAR, USA

**Keywords:** SERM, cannabinoid, CB1, CB2, inverse agonist, antagonist, G-protein coupled receptor, drug development

## Abstract

Selective estrogen receptor modulators (SERMs) are used to treat estrogen receptor (ER)-positive breast cancer and osteoporosis. Interestingly, tamoxifen and newer classes of SERMs also exhibit cytotoxic effects in cancers devoid of ERs, indicating a non-estrogenic mechanism of action. Indicative of a potential ER-independent target, reports demonstrate that tamoxifen binds to cannabinoid receptors (CBRs) with affinity in the low μM range and acts as an inverse agonist. To identify cannabinoids with improved pharmacological properties relative to tamoxifen, and further investigate the use of different SERM scaffolds for future cannabinoid drug development, this study characterized the affinity and activity of SERMs in newer structural classes at CBRs. Fourteen SERMs from five structurally distinct classes were screened for binding to human CBRs. Compounds from four of five SERM classes examined bound to CBRs. Subsequent studies fully characterized CBR affinity and activity of one compound from each class. Ospemifine (a triphenylethylene) selectively bound to CB1Rs, while bazedoxifine (an indole) bound to CB2Rs with highest affinity. Nafoxidine (a tetrahydronaphthalene) and raloxifene (RAL; a benzothiaphene) bound to CB1 and CB2Rs non-selectively. All four compounds acted as inverse agonists at CB1 and CB2Rs, reducing basal G-protein activity with IC_50_ values in the nM to low μM range. Ospemifine, bazedoxifene and RAL also acted as inverse agonists to elevate basal intracellular cAMP levels in intact CHO-hCB2 cells. The four SERMs examined also acted as CB1 and CB2R antagonists in the cAMP assay, producing rightward shifts in the concentration-effect curve of the CBR agonist CP-55,940. In conclusion, newer classes of SERMs exhibit improved pharmacological characteristics (e.g., in CBR affinity and selectivity) relative to initial studies with tamoxifen, and thus suggest that different SERM scaffolds may be useful for development of safe and selective drugs acting via CBRs.

## Introduction

**FIGURE 1:**
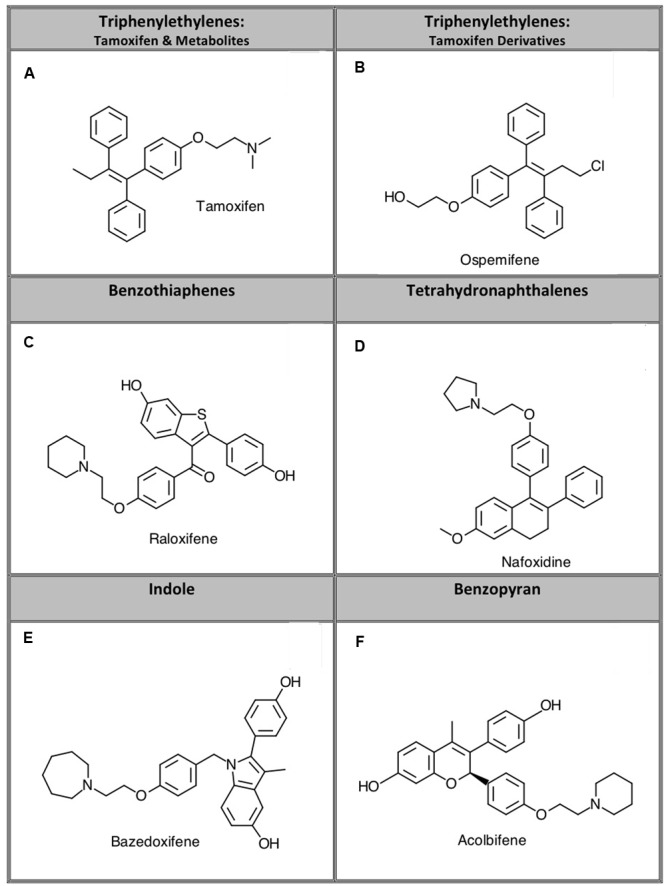
**Representative compounds from five structurally distinct SERM classes examined in this study.** Specific compounds studied were: **(A)** tamoxifen, **(B)** ospemifene, **(C)** raloxifene, **(D)** nafoxidine, **(E)** bazedoxifene, and **(F)** acolbifene.

Selective estrogen receptor modulators act as agonists or antagonists at estrogen receptors (ERs) in a tissue specific fashion (Arnott et al., 2014). SERMs are used for several therapeutic purposes including treating ER-positive breast cancer, preventing osteoporosis, and mitigating postmenopausal conditions including dysregulation of bone density and serum lipids (Maximov et al., 2013). The first SERM that was successfully used for its antiestrogenic activity to treat breast cancer was tamoxifen. Although tamoxifen acts as an ER antagonist in breast tissue, it exhibits agonist activity at ERs in the uterus, which increases the risk and incidence of endometrial cancer (Maximov et al., 2013). In addition to these undesired agonist effects in the uterus, tamoxifen can produce other adverse side effects including hot flashes, increased risk of stroke and pulmonary embolism, and ocular changes (Arnott et al., 2014). In attempt to develop drugs in this class with fewer risks and side effects than tamoxifen, additional SERMs were synthesized that exhibit different tissue-specific activity (Arnott et al., 2014). Compounds developed based on tamoxifen as a scaffold are classified as triphenylethylene SERMs. Other classes of SERMs exhibiting varying degrees of affinity for ERα and ERβ with desirable tissue specificity are grouped structurally into the benzothiophene, tetrahydronaphthalene, indole, or benzopyran classes (**Figure 1**) (Arnott et al., 2014).

Drugs in the five major classes of SERMs were originally designed to treat hormone-sensitive forms of cancers, bone disorders, and cardiovascular issues. However, many of these SERMs at higher doses have surprisingly been shown to also produce cytotoxicity in cancers devoid of the ERs (Nagahara et al., 2013). Our laboratory and others (Kumar and Song, 2013; Prather et al., 2013) have suggested that cannabinoids receptors (CBRs) might represent one potential ER-independent mechanism responsible for SERM cytotoxicity in some forms of cancer. For example, SERMs bind to the CBRs with moderate to high affinity (Kumar and Song, 2013; Prather et al., 2013), and SERMs and cannabinoids exhibit overlapping anti-proliferative, anti-angiogenic and pro-apoptotic actions.

Cannabinoids receptors are seven trans-membrane domain-spanning receptors that couple to the G_i/o_-subtype of G-proteins. There are two commonly accepted receptor subtypes: cannabinoid type-1 (CB1) and cannabinoid type-2 (CB2) (Pisanti et al., 2013). CB1 receptors are expressed in highest density in the central nervous system (CNS) but are also present in other tissues, including reproductive organs and the gastrointestinal tract (Cabral et al., 2015). CB2 receptors are also present in low levels in the nervous system, but are expressed in greatest abundance in, and modulate the function of, immune cells (Cabral et al., 2015). Ligands that bind to and modulate the activity of CBRs (e.g., cannabinoids) are structurally diverse and range from compounds that are endogenously produced (endocannabinoids), to plant-derived (phytocannabinoids) and synthesized compounds (synthetic cannabinoids). In addition to acting at CB1 and CB2 receptors, cannabinoids have also been shown to produce effects via modulation of other targets including peroxisome proliferator-activated receptors (PPAR) (Martinez et al., 2015) and transient receptor potential vanilloid type-1 (TRPV1) channels (Morgese et al., 2007). The only cannabinoids that are currently approved by the FDA for therapeutic purposes are plant-derived and act non-selectively at CB1 and CB2Rs (Pertwee, 2010). Synthetic cannabinoids, however, have been developed that act selectively at CB1 or CB2 receptors. Ligands acting via CB1 receptors primarily modulate CNS function and have been investigated for potential therapeutic uses including neuroprotection during epilepsy, Parkinson’s disease, Huntington’s disease, pain, nausea and for appetite enhancement (Pertwee, 2010; García et al., 2011; Valdeolivas et al., 2012). Development of CB2-selective cannabinoids may also prove useful for treatment of immune-related disorders by altering inflammation and cytokine levels (Xu et al., 2007; Cabral and Griffin-Thomas, 2009), cancer via anti-proliferative and anti-angiogenic effects (Vidinsky et al., 2012), and chronic neuropathic pain (Wilkerson et al., 2012).

Thus, development of cannabinoids with high affinity and selectivity for either CB1 or CB2 receptors could allow for a more targeted therapeutic approach for treatment of a variety of disease states with reduced side effects. Tamoxifen has been used safely for decades and binds non-selectively to CB1 and CB2 receptors with affinity in the low μM range (Kumar and Song, 2013; Prather et al., 2013). To identify cannabinoids with improved pharmacological characteristics relative to tamoxifen, and investigate the usefulness of SERM scaffolds for future cannabinoid drug development, the purpose of this study was to characterize the affinity and activity of SERMs in newer structural classes at CBRs.

## Materials and Methods

### Materials

All SERMs were obtained from the following commercial sources. tamoxifen and Y-134 were purchased from Tocris Bioscience (Bristol, United Kingdom). *N*-desmethyl tamoxifen, 4-hydroxy tamoxifen, endoxifen, SO_4_-tamoxifen, toremifene, 4-hydroxy toremifene, OSP, RAL, lasofoxifene, NAF, and BAZ were all obtained from Sigma Aldrich (St. Louis, MO, USA). Acolbifene was procured from AdooQ Bioscience (Irvine, CA, USA).

AM-630, AM-251, DAMGO, and WIN-55,212-2 were purchased from Tocris Bioscience. CP-55,940 was obtained from Santa Cruz Biotechnology, Inc. (Dallas, TX, USA). [^35^S]GTPγS (1250 Ci/mmol) was procured from American Radiolabeled Chemicals (St. Louis, MO, USA) and [^3^H]CP-55,940 (131.4 Ci/mmol) was purchased from PerkinElmer (Waltham, MA, USA).

All other reagents were purchased from Fisher Scientific Inc. (Pittsburgh, PA, USA). All compounds were dissolved in 100% DMSO to produce a stock concentration of 10 mM.

### Methods

#### Cell Culture

All experiments were conducted using intact cells or membranes prepared from Chinese hamster ovary (CHO) cells stably transfected with either human cannabinoid type-1 receptors (CHO-hCB1), human cannabinoid type-2 receptors (CHO-hCB2) (Shoemaker et al., 2005), or human mu opioid receptors (CHO-hMOR) (Seely et al., 2012). CHO-hCB1 cells were purchased from DiscoverRx Corporation (Fremont, CA, USA) and cultured in HAM’s F-12 K media (ATCC, Manassas, VA, USA). CHO-hCB2 and CHO-hMOR cells were cultured in DMEM (Mediatech Inc., Manassas, VA, USA). All media contained 10% Fetalplex, (Gemini Bioproducts, Sacramento, CA, USA), 0.05 IU/mL penicillin, 50 μg/mL streptomycin (Invitrogen, Carlsbad, CA, USA), and 250 μg/mL of Geneticin (G418; Sigma-Aldrich, St. Louis, MO, USA). Cells were cultured in a 37°C humidified incubator with 5% CO_2_ and harvested with PBS (10 mM)/EDTA (1 mM) when 80% confluent. All cells used were maintained between passages 4–15.

#### Membrane Preparation

Membrane homogenates were prepared for competition receptor binding and [^35^S]GTPγS binding studies by using pellets previously frozen of CHO-hCB1 or CHO-hCB2 cells as described in (Franks et al., 2014). In short, pellets were thawed on ice, combined in a 40 ml glass Dounce homogenizer and homogenized in 20 ml of cold buffer (50 mM HEPES at pH 7.4, 3 mM MgCl_2_, and 1 mM EGTA) using 10 strokes. Homogenates were then centrifuged at 40,000 × *g* for 10 min at 4°C. After discarding the supernatant, pellets were homogenized and centrifuged twice more. Final pellets were re-suspended in ice-cold 50 mM HEPES, pH 7.4, aliquoted and stored at -80°C for future use. Protein concentration was determined the same day, prior to freezing, using BCA^TM^ Protein Assay (Thermo Fisher Scientific, Waltham, MA, USA).

#### Competition Receptor Binding

Competition receptor binding was conducted by using 0.2 nM of the radioligand [^3^H]CP-55,940, a high-affinity and non-selective cannabinoid agonist as reported previously (Prather et al., 2013). The final volume of each sample was 1 ml, containing 50 mM Tris-HCl buffer (pH 7.4), 0.05% bovine serum albumin, 5 mM MgCl_2_, increasing concentrations of non-radioactive competing SERM ligands, and either 100 μg of CHO-hCB1 or 50 μg of CHO-hCB2 membrane homogenates. Non-specific binding was defined by radioactivity remaining in the presence of 1 μM of the non-radioactive CB1/CB2 agonist WIN-55,212-2. Each sample condition was performed in triplicate and allowed to reach equilibrium at room temperature for 90 min. Reactions were terminated by rapid vacuum filtration through Whatman GF/B glass fiber filters (Brandel, Inc.), followed by four 4 ml washes of ice-cold filtration buffer (50 mM Tris at pH 7.4 and 0.05% BSA). Filter punches of individual samples were placed in scintillation vials containing 4 mls of ScintiverseTM BD cocktail scintillation fluid (Fisher Scientific, Pittsburg, PA, USA). Final counts were determined using liquid scintillation spectrophotometry (Tri Carb 2100 TR Liquid Scintillation Analyzer, Packard Instrument Company, Meriden, CT, USA) after overnight incubation.

#### [^35^S]GTPγS Binding

The [^35^S]GTPγS binding assay was conducted as described previously (Prather et al., 2013) in a final volume of 1 ml by incubating 0.1 nM [^35^S]GTPγS in 20 mM HEPES with 10 mM MgCl_2_, 100 mM NaCl, 10 μM GDP, 0.1% bovine serum albumin, varying concentrations of SERM ligands, and either 25 μg of CHO-hCB2, 50 μg of CHO-hCB1 or 50 μg of CHO-hMOR membranes homogenates. Non-specific binding was defined by radioactivity remaining in the presence of 10 μM of non-radioactive GTPγS. All reactions were incubated for 30 min at 30°C, terminated by filtration through Whatman GF/B glass fiber filters (Brandel, Inc.) and followed by four 4 ml washes of ice cold filtration buffer (50 mM HEPES, pH 7.4) containing 0.1% bovine serum albumin. Filter punches of individual samples were placed in scintillation vials containing 4 mls of ScintiverseTM BD cocktail scintillation fluid (Fisher Scientific, Pittsburg, PA, USA). Final counts were determined using liquid scintillation spectrophotometry (Tri Carb 2100 TR Liquid Scintillation Analyzer, Packard Instrument Company, Meriden, CT, USA) after overnight incubation.

#### Adenylyl Cyclase Assay

Adenylyl cyclase activity was measured using intact CHO-hCB1 or CHO-hCB2 cells, similar to that previously reported (Rajasekaran et al., 2013). Briefly, cells cultured between passages 4 and 15 were seeded into 24-well plates at a concentration of 6.5 million cells per plate and incubated overnight in a humidified incubator maintained at 37°C and 5% CO_2_. Growth media was removed the next day and replaced with media containing 2.5 Ci/ml [^3^H]adenine, 0.9g/L NaCl, and 0.5 mM isobutyl-methyl-xanthine (IBMX) for 2–3 h. Radioactive media was then removed and each well treated with 0.5 ml of varying concentrations of SERM compounds in a Krebs-Ringer-HEPES solution (10 mM HEPES, 110 mM NaCl, 25 mM Glucose, 55 mM Sucrose, 5 mM KCl, 1 mM MgCl_2_, 1.8 mM CaCl_2_, pH 7.4) containing 0.5 mM IBMX and 10 μM forskolin. Plates were then floated in a 37°C water bath for 15 min before reactions were terminated by addition of 50 μL of 2.2 N HCl to each well.

For experiments to determine the ability of SERMs to antagonize the inhibition of adenylyl cyclase activity by CP-55,940, plates were seeded and incubated with [^3^H]adenine as described above. After removal of radioactivity, SERMs were preincubated with cells in Krebs-Ringer-HEPES solution for 30 min at room temperature prior to addition of increasing concentrations of CP-55,940 (10^-10^-10^-5^M). Following addition of CP-55,940, samples were further incubated at room temperature for 7 min at room temperature. Reactions were then terminated by adding 50 μL of 2.2 N HCl to each well. [^3^H]cAMP was isolated using alumina column chromatography and radioactivity quantified following addition of 10 mls of scintiverseTM BD cocktail scintillation fluid (Fisher Scientific, Pittsburg, PA, USA) by liquid scintillation spectrophotometry (Tri Carb 2100 TR Liquid Scintillation Analyzer, Packard Instrument Company, Meriden, CT, USA).

### Statistical Analysis

Statistical analyses were conducted by utilizing GraphPad Prism v6.0g (GraphPad Software, Inc.; San Diego, CA, USA). IC_50_ values from competition receptor binding curves were derived by non-linear regression. Experimental IC_50_ values were converted to K_i_ values (a measure of receptor affinity) by using the Cheng–Prusoff equation (Cheng and Prusoff, 1973). Non-linear regression analysis of concentration-effect curves was also used to determine potency (ED_50_ or IC_50_) and efficacy (E_max_ or I_max_) for modulation of GTPγS and adenylyl cyclase activity, respectively. All IC_50_, EC_50_, K_i_, and K_b_ values were converted to pEC_50_, pIC_50_, pK_i_, and pK_b_ values in order to permit use of parametric statistical analyses. For all experiments comparing three or more values, an one-way ANOVA was employed, followed by Dunnett’s or Tukey’s *post hoc* tests. For comparisons of two values, an unpaired *t*-test was utilized. To determine significant radioligand displacement or modulation of G-protein activity compared to basal levels, a one-sample *t*-test was employed.

## Results

### SERMs from Four of Five Structural Classes Exhibit Affinity for CB1 and CB2Rs

**FIGURE 2:**
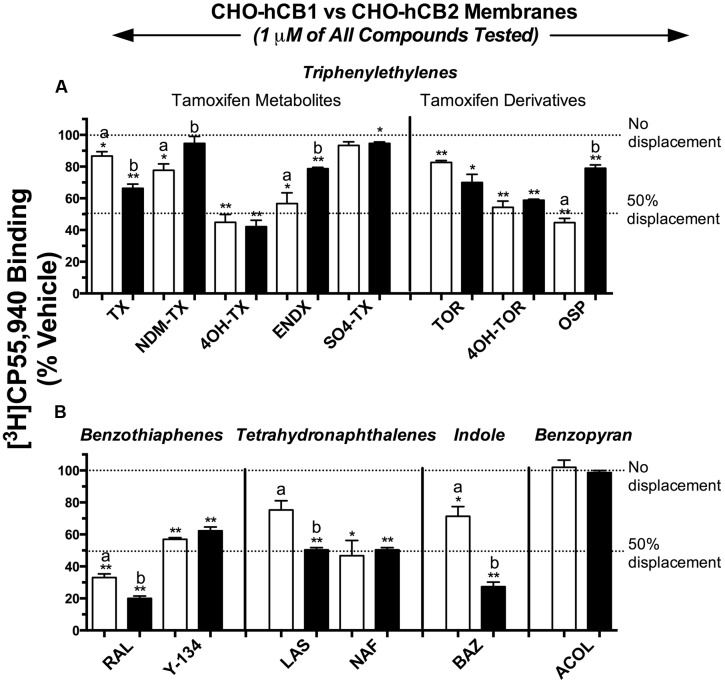
**Selective estrogen receptor modulators from four of five structural classes exhibit affinity for CB1 and CB2Rs.** Commercially available compounds from 5 structurally distinct classes were subjected to a radioligand binding screen **(A,B)**. The ability of a single 1 μM concentration of each SERM to displace 0.2 nM of the high affinity, non-selective CB1R/CB2R radioligand [^3^H]CP-55,940 from hCB1 [open bars] or hCB2 [filled bars] receptors stably expressed in CHO cells was examined. ^∗,∗∗^ Histograms that are designated by asterisks, are significantly different from 100% specific [^3^H]CP-55,940 binding (*P* < 0.05, 0.01; One-sample *t*-test). ^a,b^[^3^H]CP-55,940 binding of individual SERMs to hCB1 and hCB2 receptors designated by different letters, are significantly different (*P* < 0.05; Student’s *t*-test).

For initial comparison of potential SERM binding to CBRs, 14 commercially available compounds from 5 structurally distinct classes (**Figure 1**) were subjected to a radioligand binding screen (**Figure 2**). Specifically, the ability of a single 1 μM concentration of each SERM to displace 0.2 nM of the high affinity, non-selective CB1R/CB2R radioligand [^3^H]CP-55,940 from human CB1 (CHO-hCB1) or CB2 (CHO-hCB2) receptors stably expressed in CHO cells was examined. Based on these experimental conditions, the Cheng–Prusoff equation (Cheng and Prusoff, 1973) predicts that the concentration of a SERM producing 50% displacement of [^3^H]CP-55,940 (e.g., IC_50_) from a receptor will approximate the affinity (e.g., K_i_) of that compound for the receptor examined.

Selective estrogen receptor modulators in the triphenylethylene class (1 μM) displace [^3^H]CP-55,940 from both hCB1 and hCB2Rs by amounts ranging from little to no displacement, to as high as 58% (**Figure 2A**). Specifically for hCB1Rs (open bars), OSP, and the tamoxifen metabolites 4-hydroxy-tamoxifen (4OH-TX), endoxifen (ENDX), and 4-hydroxy-toremifene (4OH-TOR) produce the greatest amount of [^3^H]CP-55,940 displacement of approximately 50%. Concerning CB2Rs (filled bars), the triphenylethylenes produce levels of [^3^H]CP-55,940 displacement similar to that from hCB1Rs, from 5 to 58%. Importantly, data from this initial binding screen predict that while 4OH-TX and 4OH-TOR bind to hCB1 and hCB2Rs non-selectively (e.g., producing similar levels of radioligand displacement at 1 μM from both receptors), OSP exhibits some degree of selective affinity for hCB1Rs.

Based on data from the initial screen, both compounds in the benzothiophene group (**Figure 2B**) would be predicted to bind to both CBRs with moderate to high affinity in the nM range, with RAL producing 67 and 80% displacement of [^3^H]CP-55,940 from hCB1 and hCB2Rs, respectively. SERMs in the tetrahydronaphthalene class, lasofoxifene (LAS) and NAF, also apparently exhibit appreciable affinity for CBRs, with LAS predicted to bind relatively selectively to hCB2Rs. BAZ, the only SERM commercially available from the indole group, appears to exhibit higher affinity for the CB2 receptors, producing 29 and 73% displacement of [^3^H]CP-55,940 from hCB1 and hCB2Rs, respectively. Finally, acolbifene, the sole compound available for examination in the benzopyran group, fails to displace [^3^H]CP-55,940 from either hCB1 or hCB2Rs and thus would be predicted to have no affinity for CBRs.

### SERMs Act as Inverse agonists at CBRs to Modulate G-Protein Activity

**FIGURE 3:**
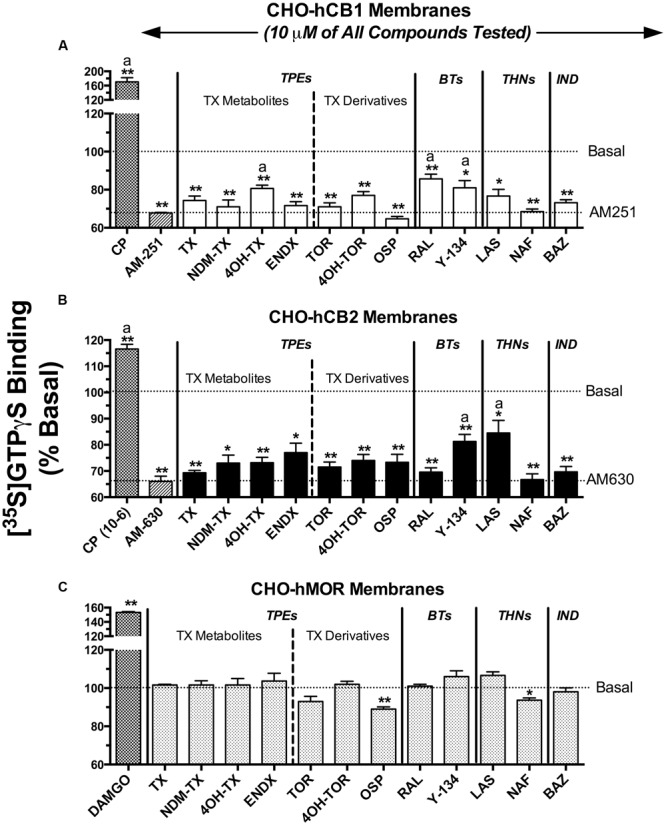
**Selective estrogen receptor modulators act as inverse agonists at CBRs to modulate G-protein activity.** SERMs predicted by initial binding studies to exhibit appreciable affinity for CBRs, were next screened for intrinsic activity at **(A)** hCB1Rs, **(B)** hCB2Rs or **(C)** hMORs by examining [^35^S]GTPγS binding in the presence or absence of a receptor-saturating concentration (10 μM) of all compounds. G-protein modulation by full agonists CP-55,940 (10 μM) and DAMGO (10 μM) was examined to serve as positive controls for activation of **(A,B)** CBRs and **(C)** MORs, respectively. G-protein modulation by the inverse agonists AM-251 and AM-630 was examined to serve as positive controls for regulation of **(A)** CB1 and **(B)** CB2R signaling, respectively. The mean ± SEM of [^35^S]GTPγS binding is presented as percent of basal G-protein activity in the presence of vehicle. ^∗,∗∗^ Histograms that are designated by asterisks, are significantly different from basal activity (*P* < 0.05, 0.01; One-sample *t*-test). ^a^[^35^S]GTPγS binding produced by individual SERMs acting at hCB1 or hCB2 receptors are significantly different that binding produced by the CBR inverse agonists AM-251 (for hCB1Rs) or AM-630 (for hCB2Rs), respectively (*P* < 0.05; Student’s *t*-test).

Selective estrogen receptor modulators that were predicted to exhibit appreciable affinity for CBRs based on initial binding studies were next screened for intrinsic activity (e.g., to determine if these SERMs act as agonists, antagonists or inverse agonists at CBRs; **Figure 3**). CBRs are coupled to G_i_/G_o_ type G-proteins. Therefore, binding of agonists to CBRs leads to G-protein activation, antagonists produce no effect, and inverse agonists reduce basal G-protein activity produced by constitutively active receptors. SERMs were screened by examining the ability of a single receptor saturating concentration (10 μM) to modulate G-protein activity in membranes prepared from CHO-hCB1 and CHO-hCB2 cells. G-protein activation was quantified using employing [^35^S]GTPγS, a non-hydrolyzable analog of GTP, that irreversibly binds to the G-proteins when activated.

When examining intrinsic activity at hCB1Rs (**Figure 3A**), as anticipated, the full hCB1/hCB2R agonist CP-55,940 (1 μM) produces a 70% increase in [^35^S]GTPγS binding above basal levels and the well-characterized CB1 inverse agonist AM-251 (10 μM) reduces basal G-protein activity over 30%. All SERMs examined, except 4OH-TX, RAL and Y-134, reduce basal G-protein activity to levels similar to that produced by the full inverse agonist AM-251. Therefore, 4OH-TX, RAL and Y-134 would be predicted to act as partial, while all other SERMs examined act as full inverse agonists at CB1Rs.

Concerning G-protein modulation by SERMs at hCB2Rs (**Figure 3B**), the full hCB1/CB2R agonist CP-55,940 (1 μM) activates G-proteins by 17%, while the full hCB2R inverse agonist AM-630 (10 μM) reduces G-protein activity by 34%. Similar to intrinsic activity observed at hCB1Rs, all SERMs from the four classes examined act as full inverse agonists, with only Y-134 and LAS exhibiting partial inverse agonist activity.

To demonstrate that the observed modulation of G-protein activity by SERMs in transfected CHO cells occurs due to action at CBRs, [^35^S]GTPγS binding assays were also conducted in membranes prepared from CHO cells devoid of cannabinoid receptors, but stably expressing mu-opioid receptors as a positive control (CHO-hMOR) (**Figure 3C**). As anticipated, the full mu-opioid agonist DAMGO increases G-protein activity by 53%. In marked contrast to that observed in CHO-hCB1 and CHO-hCB2 membranes, in CHO-hMOR membranes all SERMs except OSP do not alter basal G-protein activity. Although OSP does decrease G-protein activity by 11% in CHO cells not expressing CBRs, this SERM reduces basal G-protein activity to a much greater level (e.g., ∼30%) in CHO cells expressing CBRs. Taken collectively, these data strongly indicate that reduction in G-protein activity produced by all SERMs screened in CHO-hCB1 and CHO-hCB2 cells occurs specifically due to interaction with CBRs.

### SERMs Bind With High Affinity (e.g., K_i_ Values) and Differential Selectivity to CB1 and CB2Rs

To more fully characterize SERMs from different structural classes, one compound from each class was selected based on distinctive characteristics identified by the initial binding screen. For example, the triphenylethylene OSP and the indole BAZ were selected due to potential selective affinity for hCB1 and hCB2Rs, respectively. Although the benzothiophene RAL and tetrahydronaphthalene NAF appear to bind non-selectively to hCB1 and hCB2Rs, both were selected for further analysis due to predicted high affinity for CBRs based on the binding screen. The benzopyran acolbifene was not selected for further studies because 1 μM of this SERM did not produce any displacement of [^3^H]CP-55,940 from either CBR.

**FIGURE 4:**
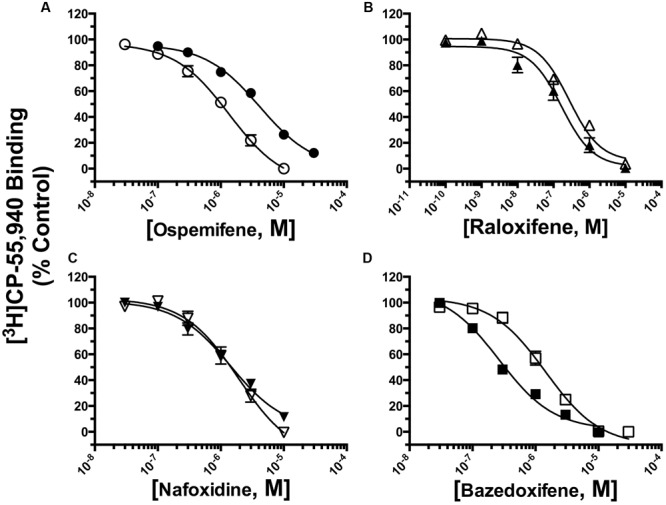
**Selective estrogen receptor modulators bind with high affinity and differential selectivity to CB1 and CB2Rs.** A measure of affinity (K_i_) of **(A)** OSP, **(B)** RAL, **(C)** NAF, and **(D)** BAZ for CB1 and CB2Rs was obtained by conducting competition binding studies, employing 0.2 nM [^3^H]-CP-55,940 and increasing concentrations of SERMs (10^-10^ to 10^-5^M). K_i_ values (mean ± SEM) were derived from non-linear regression analysis of the curves shown by using the Cheng–Prusoff equation (Cheng and Prusoff, 1973). Individual K_i_ values and statistical analysis of pK_i_ values are presented in **Table 1**. Filled symbols represent binding of SERMs to CB2Rs, open symbols represent binding of SERMs to CB2Rs.

The affinity (K_i_) of each compound was determined by full competition receptor binding curves (**Figure 4**) employing the CB1/CB2R radioligand [^3^H]CP-55,940 in membranes prepared from CHO-hCB1 and CHO-hCB2 cells. K_i_ values were derived from experimental IC_50_ values employing the Cheng–Prusoff equation (Cheng and Prusoff, 1973) and are presented in **Table 1**. For hCB1Rs, RAL exhibits the highest affinity with a K_i_ value of 210 nM, while NAF, OSP and BAZ bind to hCB1Rs with moderate affinity in the sub-micromolar range. Considering CB2Rs, RAL and BAZ exhibit high affinity with K_i_ values in the low nM range (240 and 254 nM, respectively). NAF and OSP bind to hCB2Rs with statistically lower affinity (1–4 μM) than RAL or BAZ. As predicted by the initial binding screen (**Figure 2**), OSP binds relatively selectively to hCB1Rs with a CB1/CB2 ratio of 0.20, while BAZ exhibits selectivity for hCB2Rs with a CB1/CB2 ratio of 3.29. Also as predicted, RAL and NAF lacked any selectivity and bound to both hCB1and hCB2Rs with similar affinity.

**Table 1:** Competition binding between ospemifene, raloxifene, nafoxidine, and bazedoxifene and the CBR agonist [^3^H]CP-55,940 employing CHO-hCB1 and CHO-hCB2 membranes.

Drug	[^3^H]CP-55,940 Binding						
	CHO-hCB1			CHO-hCB2			Selectivity
	K_i_ (nM)	pK_i_	N	K_i_ (nM)	pK_i_	N	(CB1/CB2)
OSP	753	6.123 ± 0.060^a^	5	3715	5.430 ± 0.033^a,∗∗^	3	0.20
RAL	210	6.677 ± 0.063^b^	4	240	6.620 ± 0.037^b^	3	0.88
NAF	957	6.019 ± 0.084^a^	3	1300	5.886 ± 0.044^c^	3	0.74
BAZ	836	6.078 ± 0.066^a^	5	254	6.595 ± 0.023^b,∗∗^	5	3.29

*^a,b,c^pKi values designated by different letters are significantly different from values in the same column; *P* < 0.05, one-way ANOVA, Tukey *Post-hoc* test. ^∗∗^ pKi values for individual SERMs examined at hCB2 receptors are significantly different from pKi values for same compounds at hCB1 receptors; *P* < 0.01, student’s *t*-test.*

Collectively, these data suggest that the different SERM scaffolds can be modified to develop non-selective and selective hCB1 and hCB2R ligands with high affinity.

### SERMs Modulate G-Protein Activity With Potencies Predicted by Affinity for hCB1 and hCB2Rs

**FIGURE 5:**
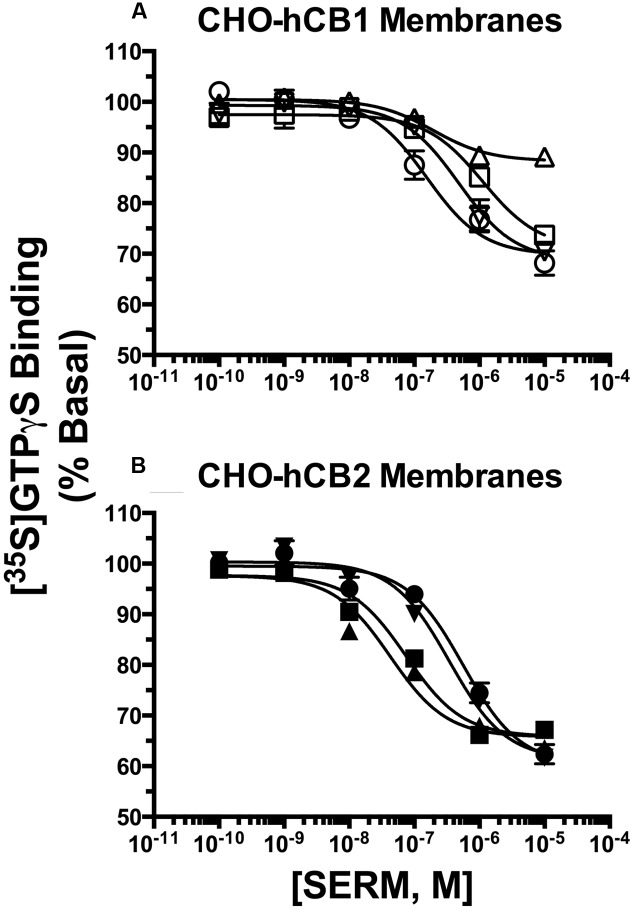
**Selective estrogen receptor modulators modulate G-protein activity with potencies predicted by affinity for hCB1 and hCB2Rs.** The ability of OSP (circles), RAL (upward triangles), NAF (downward triangles) and BAZ (squares) to modulate G-protein activity via **(A)** CB1Rs and **(B)** CB2Rs was evaluated by examining [^35^S]GTPγS binding in the presence increasing concentrations (10^-10^ to 10^-5^M) of all SERMs. All IC_50_ and I_MAX_ values (mean ± SEM) were derived from non-linear regression analysis of the curves shown and are presented in **Table 2** with statistical analysis.

To further characterize intrinsic activity at CBRs, full concentration-effect curves were conducted to determine the potency (IC_50_) and efficacy (I_max_) for SERM modulation of G-protein activity by hCB1 and hCB2Rs (**Figure 5**; **Table 2**). Concerning hCB1Rs (**Figure 5A**), OSP and RAL potently reduce basal G-protein activity with IC_50_ values of 170 and 143 nM. Both SERMs are significantly more potent than either NAF or BAZ. Importantly, the rank order of potency for CB1R-mediated G-protein modulation by these SERMs is identical to the rank order of their affinity for CB1Rs (e.g., RAL > OSP > NAF = BAZ). When considering efficacy, as predicted by the initial intrinsic efficacy screen (**Figure 3**), all SERMs except RAL acted as full inverse agonists at hCB1Rs.

**Table 2:** Modulation of GTPγS binding by SERMs employing CHO-hCB1 and CHO-hCB2 membranes.

Drug	GTPγS Binding (% Basal)						
	CHO-hCB1				CHO-hCB2			
	IC_50_ (nM)	pIC_50_	I_MAX_ (%)	N	IC_50_ (nM)	pIC_50_	I_MAX_ (%)	N
OSP	170	6.769 ± 0.147^a^	30.3 ± 2.0^a^	3	558	6.253 ± 0.117^a,∗∗^	39.7 ± 2.4^a,∗∗^	3
RAL	143	6.846 ± 0.174^a^	11.7 ± 4.7^b^	3	87.1	7.060 ± 0.190^b^	34.3 ± 2.2^b,∗∗^	3
NAF	524	6.281 ± 0.105^b^	31.7 ± 1.5^a^	3	344	6.463 ± 0.073^a^	38.7 ± 1.2^a,b,∗∗^	3
BAZ	1164	5.934 ± 0.146^b^	29.7 ± 1.9^a^	3	71.6	7.145 ± 0.085^b,∗∗^	34.0 ± 1.0^b,∗^	3

*^a,b^pIC_50_ or I_MAX_ values designated by different letters are significantly different from values in the same column; *P* < 0.05, one-way ANOVA, Tukey *Post hoc* test. ^∗,∗∗^pIC_50_ or I_MAX_ values for individual SERMs examined at hCB2 receptors are significantly different from pIC_50_ or I_MAX_ values for same compounds at hCB1 receptors; *P* < 0.05, 0.01, student’s *t*-test.*

When examining activity at hCB2Rs (**Figure 5B**), BAZ and RAL more potently (IC_50_ = 71.6 and 87.1 nM, respectively) reduce basal G-protein activity when compared to OSP or NAF. As observed for hCB1Rs, the rank order of potency for CB2R-mediated G-protein modulation by these SERMs is identical to the rank order of their affinity for CB2Rs (e.g., RAL = BAZ > NAF > OSP). All SERMs were relatively equally efficacious, maximally reducing G-protein activity via hCB2Rs with I_max_ values of approximately 40%.

### SERMs Also Act as Inverse CB2 Agonists to Modulate of Adenylyl Cyclase Activity

**FIGURE 6:**
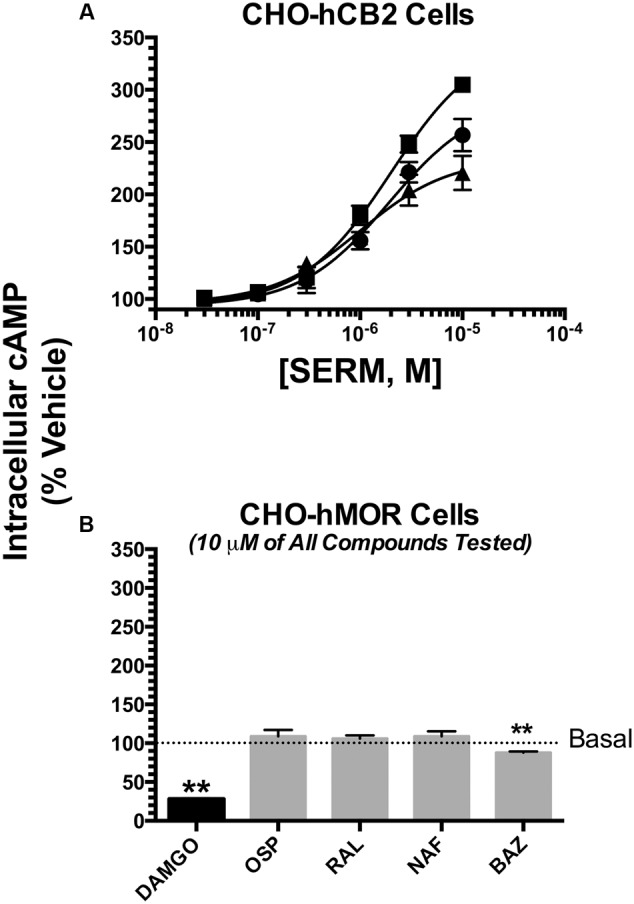
**Selective estrogen receptor modulators also act as inverse CB2 agonists to modulate of adenylyl cyclase activity.** The potency (EC_50_) and efficacy (E_MAX_) for modulation of forskolin-stimulated adenylyl cyclase activity was evaluated by analyzing concentration-effect curves (10^-8^ to 10^-5^M) for OSP (circles), RAL (triangles) and BAZ (squares) in intact CHO-hCB2 cells **(A)**. All IC_50_ and I_MAX_ values (mean ± SEM) were derived from non-linear regression analysis of the curves shown and are presented in **Table 3** with statistical analysis. In **(B)**, CHO-hMOR cells were employed as a positive control, and adenylyl cyclase activity was measured in the presence of a receptor saturating concentration (10 μM) of the mu-opioid agonist DAMGO, or the SERMs OSP, RAL, NAF, and BAZ. The mean ± SEM of cAMP production is presented as percent of basal adenylyl cyclase activity in the presence of vehicle. ^∗∗^Bar graphs in **(B)** that are designated by asterisks, are significantly different from basal activity (*P* < 0.01; One-sample *t*-test).

To provide a second measure of intrinsic activity, the ability of SERMs to modulate intracellular levels of cAMP in intact cells was examined (**Figure 6**). CBRs couple to G_i_/G_o_-proteins, and thus cannabinoid agonists inhibit activity of the downstream intracellular effector adenylyl cyclase, reducing intracellular cAMP levels. Cannabinoid antagonists do not affect cAMP levels, and inverse agonists increase cAMP levels due to inhibition of constitutively active CBRs.

Modulation of adenylyl cyclase activity by hCB1Rs could not be examined in CHO-hCB1 cells because all SERMs tested, as well as the well established hCB1R full inverse agonist AM-251 (with concentrations as high as 10 μM) do not alter basal cAMP levels (data not shown). This indicates that the CHO-hCB1 cell line likely does not express a sufficient density of constitutively active CB1Rs to detect inverse agonism in this assay.

However, as predicted by intrinsic activity observed for hCB2R modulation of G-protein activity (**Figure 5B**), all SERMs examined similarly act as inverse agonists at CB2Rs, producing robust increases intracellular cAMP levels (**Figure 6A**; **Table 3**). BAZ, OSP and RAL elevate cAMP with E_max_ values of 352, 279 and 234%, respectively. Very interestingly, all SERMs examined are more efficacious when compared to the well-established full inverse agonist AM-630, which increases cAMP levels in CHO-hCB2 cells by only 214 ± 6.4% (**Table 3**). When considering potency, RAL increases cAMP levels most potently with an EC_50_ of 873 nM, while the potency of OSP and BAZ is only in the low micromolar range. As anticipated, based on lower affinity for hCB2Rs, all SERMs were also less potent than AM-630 in this assay. Quantification of the intrinsic activity of NAF for regulation of adenylyl cyclase activity in CHO-hCB2 cells could not be determined due to solubility limitations that precluded examination of concentrations high enough for calculation of accurate EC_50_ and E_max_ values. Modulation of adenylyl cyclase activity in CHO-hCB2 cells by SERMs is mediated by hCB2Rs because basal cAMP levels in CHO-hMOR cells, that do not express hCB2Rs, are only slightly altered by one SERM (e.g., BAZ) (**Figure 6B**).

**Table 3:** Modulation of adenylyl cyclase activity by SERMs in intact CHO-hCB2 cells.

Drug	Intracellular [^3^H]cAMP			
	EC_50_ (nM)	pEC_50_	E_MAX_ (%)	N
OSP	1445	5.840 ± 0.096^a,b^	279 ± 17.0^a^	3
RAL	873	6.059 ± 0.088^a^	234 ± 21.0^b^	3
BAZ	1982	5.703 ± 0.105^b^	352 ± 14.0^c^	3
AM-630^†^	398	6.40 ± 0.096^c^	214 ± 6.5^b^	4

*^a,b,c^pEC_50_ and E_MAX_ values designated by different letters are significantly different from values in the same column; *P* < 0.05, one-way ANOVA, Tukey *Post-hoc* test. ^†^Values obtained from Ford et al. (2016).*

### SERMs Produce Surmountable Antagonism of CP-55,940 Inhibition of Adenylyl Cyclase in CHO-hCB1 Cells

**FIGURE 7:**
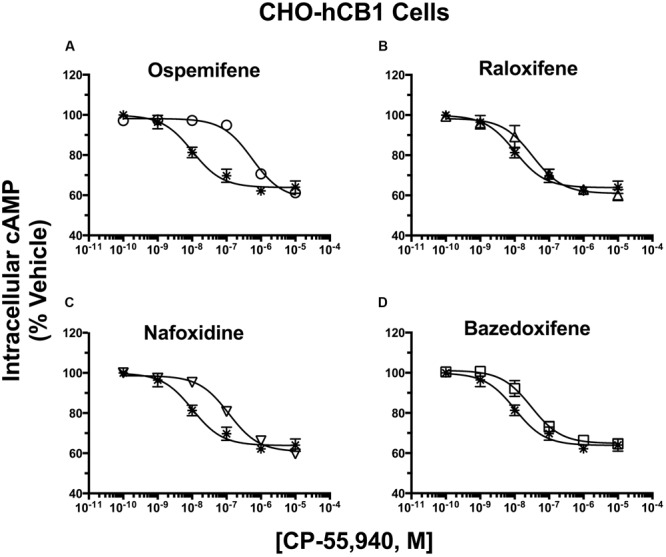
**Selective estrogen receptor modulators produce surmountable antagonism of CP-55,940 inhibition of adenylyl cyclase in CHO-hCB1 cells.** CHO-hCB1 cells were pre-incubated for 30 min with receptor saturating concentrations of individual SERMs and were subsequently co-incubated for 7 min with increasing concentrations of CP-55,940. Measurements of CP-55,940 effects alone on potency (IC_50_) and efficacy (I_MAX_) of intracellular cAMP were obtained and were compared to the shifts in IC_50_ and I_MAX_ values observed by co-incubation with individual SERMS: **(A)** Ospemifene, **(B)** Raloxifene, **(C)** Nafoxidine, and **(D)** Bazedoxifene. All IC_50_, I_MAX_ and K_b_ values (mean ± SEM) were derived from non-linear regression analysis of the curves shown and are presented in **Table 4** with statistical analysis. Asterisk symbols represent the concentration-effect curve for CP-55,940 alone, while open symbols the action of CP-55,940 in the presence of the SERM indicated.

**FIGURE 8:**
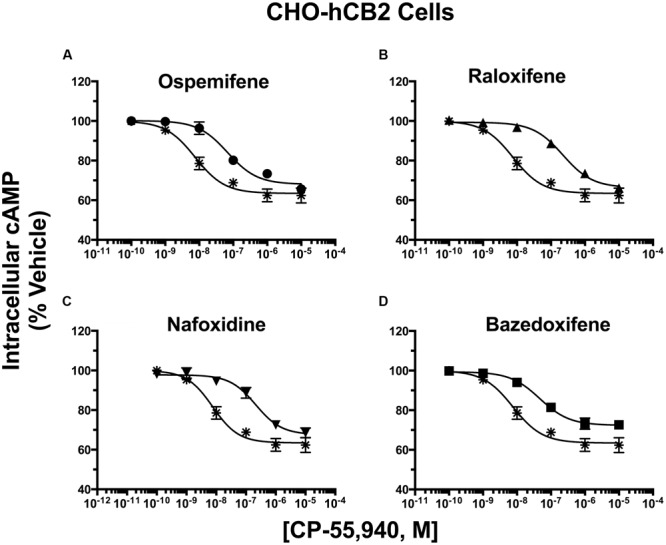
**Selective estrogen receptor modulators produce surmountable and insurmountable antagonism of CP-55,940 inhibition of adenylyl cyclase activity in CHO-hCB2 cells.** CHO-hCB2 cells were pre-incubated for 30 min with receptor saturating concentrations of individual SERMs and were subsequently co-incubated for 7 min with increasing concentrations of CP-55,940. Measurements of CP-55,940 effects alone on potency (IC_50_) and efficacy (I_MAX_) of intracellular cAMP were obtained and were compared to the shifts in IC_50_ and I_MAX_ values observed by co-incubation with individual SERMS: **(A)** Ospemifene, **(B)** Raloxifene, **(C)** Nafoxidine, and **(D)** Bazedoxifene. All IC_50_, I_MAX_, and K_b_ values (mean ± SEM) were derived from non-linear regression analysis of the curves shown and are presented in **Table 5** with statistical analysis. Asterisk symbols represent the concentration-effect curve for CP-55,940 alone, while filled symbols the action of CP-55,940 in the presence of the SERM indicated.

To demonstrate potential pharmacological relevance and provide additional support that SERMs act as CBR inverse agonists/antagonists, antagonist dissociation constants (e.g., K_b_ values) were determined by examining the effect of SERM co-incubation on the potency (IC_50_) and efficacy (I_max_) of CP-55,940 modulation of adenylyl cyclase activity in intact CHO-hCB1 and CHO-hCB2 cells (**Figures 7** and **8**). IC_50_ and K_b_ values were converted to pIC_50_ and pK_b_ values (pIC_50_ = -Log[IC_50_] or pK_b_ = -Log[K_b_], respectively) to allow use of parametric statistics for comparison.

In absence of co-incubation with any SERM, the CB1/CB2R full agonist CP-55,940 produces a concentration-dependent decrease in cAMP production with a potency (IC_50_) of 10.4 nM and an efficacy (I_max_) of 36.0% in intact CHO-hCB1 cells (**Figure 7**; **Table 4**). To obtain a near maximal response, a receptor saturating concentration of each SERM (based on receptor affinity) was selected for co-incubation with CP-55,940. Co-incubation with all SERMs produces a significant decrease in potency, but not efficacy, of CP-55,940-mediated inhibition of adenylyl cyclase activity (**Table 4**). Graphically, this is observed as a parallel rightward shift in the concentration-effect curve for CP-55,940 (**Figures 7A–D**), and indicates that all SERMs act as surmountable CB1R antagonists in this assay. The degree of shift produced by co-incubation was used to calculate a K_b_ value for each SERM (the antagonist dissociation constant), a quantifiable measure of antagonism. The high affinity and well characterized CB1R inverse agonist/antagonist AM-281, produces a 16-fold decrease in potency of CP-55,940 with a calculated a K_b_ value of 155 nM (**Table 4**). Although all SERMs clearly act as surmountable hCB1R antagonists, the rank order of K_b_ values for SERM antagonism of CP-55,940 inhibition of adenylyl cyclase activity via hCB1Rs (e.g., OSP > RAL = NAF > >BAZ; **Table 4**) fails to completely correlate with the rank order of affinity of these compounds for hCB1Rs (e.g., RAL > OSP = NAF = BAZ; **Table 1**). For example, because RAL exhibits highest affinity (e.g., lowest K_i_ value) for hCB1Rs, this SERM would also be predicted to act as the antagonist with the lowest K_b_ value. However, this was not observed, with OSP instead exhibiting the lowest K_b_ value of 210 nM (**Table 4**), while displaying only moderate affinity for CB1Rs of 753 nM (**Table 1**).

**Table 4:** Selective estrogen receptor modulator antagonism of CP-55,940 inhibition of adenylyl cyclase activity in intact CHO-hCB1 cells.

Drug	Intracellular [^3^H]cAMP						
	Pre-Incubation	IC_50_ (nM)	pIC_50_	I_MAX_ (%)	K_b_ (nM)	pK_b_	N
CP-55,940	——	10.4	7.982 ± 0.125	36.0 ± 2.2	——	——	4
+OSP	10 μM	614	6.212 ± 0.078^∗∗^	43.8 ± 2.7	173	6.762 ± 0.122^a^	4
+RAL	2 μM	26.6	7.575 ± 0.200^∗^	38.7 ± 1.9	1122	5.950 ± 0.062^b^	3
+NAF	10 μM	102	6.990 ± 0.118^∗∗^	38.3 ± 1.7	1164	5.934 ± 0.161^b^	4
+BAZ	10 μM	30.5	7.515 ± 0.176^∗∗^	35.3 ± 0.7	4519	5.345 ± 0.044^c^	3
+AM-281^†^	1 μM	166	6.78 ± 0.053^∗∗^	35.7 ± 0.9	155	6.810 ± 0.061^a^	3

*^∗,∗∗^pIC_50_ and I_MAX_ values are significantly different from the pIC_50_ and I_MAX_ value for CP-55,940; *P* < 0.05, 0.01, one-way ANOVA, Dunnett’s *Post hoc* test. ^a,b,c^pK_b_ values designated by different letters are significantly different from values in the same column; *P* < 0.05, one-way ANOVA, Tukey *Post hoc* test. ^†^Values obtained from Ford et al. (2016).*

### SERMs Produce Surmountable and Insurmountable Antagonism of CP-55,940 Inhibition of Adenylyl Cyclase Activity in CHO-hCB2 Cells

The same co-incubation strategy was applied to determine K_b_ values for SERMs acting via hCB2Rs (**Figure 8**; **Table 5**). When incubated alone with intact CHO-hCB2 cells, the full CB1/CB2R agonist CP-55,940 inhibited adenylyl cyclase activity in a concentration-dependent manner with a potency (IC_50_) of 8.62 nM and an efficacy of (I_max_) of 36.1%. As anticipated, co-incubation with the established hCB2R inverse agonist/antagonist AM-630 resulted in a 33-fold parallel shift-to-the-right in the concentration-effect curve of CP-55,940, resulting in a calculated K_b_ value of 55.4 nM (**Table 5**).

**Table 5:** Selective estrogen receptor modulator antagonism of CP-55,940 inhibition of adenylyl cyclase activity in inact CHO-hCB2 cells.

Drug	Intracellular [^3^H]cAMP					
	Pre-Incubation	IC_50_ (nM)	pIC_50_	I_MAX_ (%)	K_b_ (nM)	pK_b_	N
CP-55,940	——	8.62	8.246 ± 0.151	36.1 ± 2.83	——	——	8
+OSP	10 μM	65.9	7.218 ± 0.124^∗∗^	31.7 ± 2.3	1256	5.901 ± 0.222^a^	3
+RAL	2 μM	229	6.713 ± 0.100^∗∗^	33.0 ± 1.3	66.5	7.177 ± 0.242^b^	6
+NAF	10 μM	226	6.691 ± 0.145^∗∗^	32.3 ± 2.3	337	6.473 ± 0.187^c^	3
+BAZ	2 μM	52.9	7.309 ± 0.095^∗∗^	28.8 ± 3.0^∗∗^	——	——	4
+AM-630^†^	1 μM	288	6.540 ± 0.060^∗∗^	31.7 ± 0.3	55.4	7.26 ± .064^b^	3

*^∗∗^pIC_50_ and I_MAX_ values are significantly different from the pIC_50_ and I_MAX_ value for CP-55,940; *P* < 0.01, one-way ANOVA, Dunnett’s *Post hoc* test. ^a,b,c^pK_b_ values designated by different letters are significantly different from values in the same column; *P* < 0.05, one-way ANOVA, Tukey *Post hoc* test. ^†^Values obtained from Ford et al. (2016).*

As observed with hCB1Rs, SERM co-incubation in CHO-hCB2 cells also results in significant decreases in the potency (e.g., higher IC_50_ values) of CP-55,940 to inhibit adenylyl cyclase activity (**Table 5**), as reflected by shifts to the right in all concentration-effect curves (**Figure 8**). Three of the four SERMs examined (RAL, NAF and OSP) also do not alter the efficacy (e.g., I_max_ value) of CP-55,940, indicating that these compounds likely act as surmountable antagonists at hCB2Rs. Furthermore, unlike that observed for hCB1Rs, the rank order of K_b_ values for SERM-antagonism of adenylyl cyclase activity by CP-55,940 via hCB2Rs, correlates well with the rank order of affinity of these compounds for hCB2Rs (e.g., RAL > NAF > OSP; **Table 1**). Interestingly, co-incubation with BAZ (a SERM in the indole structural class), significantly decreases the efficacy (I_max_) of CP-55,940 in this assay, suggesting this compound appears to act as an insurmountable antagonist at hCB2Rs. The K_b_ value for BAZ acting via hCB2Rs could not be determined because insurmountable antagonism violates the assumption of competitive antagonism required for K_b_ calculation.

## Discussion

The major findings of this study are that compounds within four of five structurally distinct classes of SERMs bind to CBRs with moderate to high affinity, exhibit differential CB1 and CB2 selectivity, and act as partial or full inverse agonists. Although two initial reports have shown that a limited number SERMs bind to CB2Rs (Kumar and Song, 2013, 2014; Prather et al., 2013), no studies other than our initial observations with tamoxifen (Prather et al., 2013) have investigated whether these compounds also exhibit potential affinity and activity at CB1Rs, or if additional SERMs might exhibit higher affinity and/or selectivity for binding to CB1 or CB2Rs. Observations reported here demonstrate that newer classes of SERMs exhibit improved pharmacological characteristics (e.g., in CBR affinity and selectivity) relative to these initial studies, and thus suggest that several of the distinct SERM scaffolds may be useful for future development of safe and selective drugs acting via CBRs.

Knowledge that SERMs act via CBRs at pharmacologically relevant concentrations has potential therapeutic significance for several reasons. First, although the most well established mechanism of action for SERMs occurs through modulation of ERs (Arnott et al., 2014; Ellis et al., 2015), interaction with non-ER targets such as CBRs may expand therapeutic actions of these compounds and explain currently observed anti-cancer or anti-oxidant properties in tissues not expressing ERs (Perry et al., 1995; Moreira et al., 2004; Arnott et al., 2014). For example, tamoxifen exhibits anti-tumor activity in several types of cancer devoid of ERs, including pancreatic (Tomao et al., 2002), glioma (Mastronardi et al., 1998) and melanoma (Beguerie et al., 2010). Since both SERMs and cannabinoids reduce tumor angiogenesis by inhibiting VEGF (Blázquez et al., 2003; Garvin and Dabrosin, 2003), perhaps this and other shared mechanisms of action might be due to SERM interaction with CBRs. Second, in addition to serving as potential ER-independent targets, CB2R expression is significantly increased in several forms of ER positive breast cancer, and is negatively correlated to patient survival (Pérez-Gómez et al., 2015). Therefore, it is possible that SERMs exhibiting a dual mechanism of action to both antagonize ER function and act as CBR inverse agonists might exhibit superior therapy and lead to development of a novel class of specialized SERMs for use in personalized cancer therapy. Third, in addition to treatment of osteoporosis and prevention of breast cancer in post-menopausal women (Gizzo et al., 2013), RAL has been shown to produce neuroprotective (Ishihara et al., 2015) and beneficial cardiometabolic effects (Dayspring et al., 2006), as well as improve cognition in both male and female schizophrenic patients (Kindler et al., 2015). Although involvement of ERs has been implicated for mediation of several of these effects (Khan, 2016), CB1 antagonists/inverse agonists similarly improve neurocognitive symptoms in schizophrenics (Roser et al., 2010), are neuroprotective (Sommer et al., 2006) and improve cardiometabolic measures (Ginsberg and Woods, 2009). It is therefore tempting to speculate that dual action of RAL at ERs and CBRs may contribute to these potentially important therapeutic effects, and suggest a need for further investigation and development of drugs with similar mechanisms of action. Finally, given that cannabinoid actions can also be mediated by receptors other than CB1 and CB2 (Morgese et al., 2007; Martinez et al., 2015), future studies examining the affinity of SERMs for such additional targets including PPAR and/or TRPV1 channels might be informative.

Although dual action of SERMs at ERs and CBRs might be therapeutically beneficial in some situations, it is possible that interaction CBRs might also contribute to some of the adverse effects observed with this class of compounds. For example, tamoxifen use in humans (Yang et al., 2013) and research of CBR inverse agonists demonstrate that both groups of compounds increase bone mineralization, sensitivity to nociception and may result in depression (Idris and Ralston, 2010; Buggy et al., 2011; Nazarali and Narod, 2014; Azizi-Malekabadi et al., 2015). Furthermore, endogenously produced cannabinoids (e.g., endocannabinoids) are important modulators of cerebral blood flow (Benyo et al., 2016) and agonist activation of CB2Rs reduces infarct volume and improves functional outcome in experimental stroke (England et al., 2015). Antagonism of CB2R function by SERMs might thus contribute to the increased stroke incidence sometimes observed with this class of drugs (Rizzoli et al., 2011). In any case, additional research will be required to clearly delineate the participation of CBRs in both potential therapeutic and adverse effects of SERMs.

Another, and perhaps most important, implication from findings presented here is the potential for development of a novel class of drugs based on different SERM scaffolds that act selectively via CBRs. As an initial step toward development of CBR selective SERM-based drugs, new compounds must be designed to lack affinity for ERs. Interestingly, such studies to reduce ER affinity while maintaining anti-cancer activity of SERMs in the triphenylethylene class, as a means to discover novel anti-cancer targets, are already underway (Guo et al., 2013b). For example, several tamoxifen analogs, designated as the ridaifen compounds, retain growth inhibition in multiple cancer cell lines similar to that of tamoxifen, while totally lacking affinity for ERs (Guo et al., 2013a,b). One ridaifen compound in particular (ridaifen-B) induces autophagy in a human T-cell lymphoma cell line devoid of estrogen receptors (Nagahara et al., 2013), implicating a novel non-ER target for this class of compounds. Importantly, ridaifen compounds exhibit growth-inhibitory effects in many types of cancer that are similarly sensitive to cannabinoids, such as ER-negative breast cancer, gliomas, lung carcinoma, prostate cancer and leukemia/lymphoma (McKallip et al., 2006; Chakravarti et al., 2014; Nabissi et al., 2015). Cannabinoids produce efficacious anti-tumor activity by a variety of mechanisms, including inhibition of proliferation, induction of apoptosis, and inhibition of angiogenesis (Chakravarti et al., 2014). Collectively, these studies suggest that high affinity selective cannabinoids based on the triphenylethylene SERM scaffold, lacking ER affinity, might represent a novel class of drugs used to treat cancer that act via an ER-independent mechanism of action. In addition to anti-cancer activity, pre-clinical and clinical studies of CB1R inverse agonists have shown that drugs with this mechanism of action reduce appetite, body weight, insulin resistance, and hepatic steatosis, while CB2R inverse agonists act as potent and efficacious anti-inflammatory agents in a variety of disease states (Tam et al., 2012; Presley et al., 2015). Therefore, development of novel SERM-based selective CBR inverse agonists, lacking ER affinity, may exhibit improved safety relative to currently available compounds acting via CBRs.

Despite the limited number of compounds available for examination in the present study for each structural class, some potential structural activity relationships may be worth noting. All groups have a degree of structural likeness, consisting of three planar benzyl rings providing similarity to estrogen (Maximov et al., 2010) (**Figure 1**). Compounds in each class also contain a chemical moiety unique to each structural class that may participate in binding to CBRs, providing enhanced affinity, selectivity and/or activity. For example, all compounds in the triphenylethylene class contain a polar R-group stemming from a phenol and exhibit only moderate affinity for CBRs. However, SERMs in the benzothiophene, indole and tetrahydronaphthalene class possess chemical moieties present in recent designer synthetic cannabinoids (Debruyne and Le Boisselier, 2015; Thaxton et al., 2015), and exhibit moderate to high affinity for CBRs. The chemically distinct second generation SERM RAL, containing a polyhydroxy phenol benzothiopene group, exhibits highest, but similar affinity (∼200 nM) for both hCB1 and hCB2Rs. In contrast, altering the ER core binding domain by introduction of a 2-phenyl-3-methyl indole contained in the third generation SERM BAZ maintains high affinity for hCB2Rs, while significantly decreasing hCB1R affinity. Finally, removal of the 2-dimethylamino-ethoxy moiety of tamoxifen to produce OSP, apparently increases affinity for CB1, but not CB2Rs. Although current information concerning structural activity relationships for SERM interaction with CBRs is limited, observations from this and future studies may form the basis for design of more comprehensive medicinal chemistry studies to systematically optimize affinity and potency of SERMs acting at CB1 and/or CB2Rs.

In summary, this study characterized the affinity and activity of SERMs in newer structural classes at CBRs to identify cannabinoids with improved pharmacological properties relative to initial studies with tamoxifen. It was found that newer classes of SERMs bind to CBRs with higher affinity and exhibit differential selectivity than tamoxifen, while exhibiting similar inverse agonist activity. Therefore, different SERM scaffolds may be useful for development of selective and non-selective drugs acting via CBRs for treatment of cancer and a variety of other disease states.

## Author Contributions

LF and BF acquired, analyzed, and interpreted data, as well as assisted in designing and conception of methodology and experiments. PP did the majority of analysis, design and conception of the study. PP and LF primarily wrote the manuscript with significant input from BF. All authors are willing to approve final versions and agree to be accountable for accuracy and integrity of this work.

## Conflict of Interest Statement

The authors declare that the research was conducted in the absence of any commercial or financial relationships that could be construed as a potential conflict of interest.

## Abbreviations

BAZ: bazedoxifene
CB1R: Cannabinoid Receptor Type 1
CB2R: Cannabinoid Receptor Type 2
ER: estrogen receptor
NAF: nafoxidine
OSP: ospemifene
RAL: raloxifene
SERM: selective estrogen receptor modulator
